# Circulating Anti-Endothelial Cell Antibodies in Patients with Geographic Atrophy Related to Dry Age-Related Macular Degeneration

**DOI:** 10.3390/medicina60050810

**Published:** 2024-05-15

**Authors:** Katarzyna Żuber-Łaskawiec, Joanna Wilańska, Izabella Karska-Basta, Weronika Pociej-Marciak, Bożena Romanowska-Dixon, Marek Sanak, Agnieszka Kubicka-Trząska

**Affiliations:** 1 Chair of Ophthalmology, Faculty of Medicine, Medical College, Jagiellonian University, Kopernika Str. 38, 31-501 Krakow, Poland; katarzyna.zuber-laskawiec@uj.edu.pl (K.Ż.-Ł.);; 2 Clinic of Ophthalmology and Ocular Oncology, University Hospital, Kopernika Str. 38, 31-501 Krakow, Poland; 3 Department of Molecular Biology and Clinical Genetics, II Chair of Internal Medicine, Medical College, Faculty of Medicine, Jagiellonian University, Skawińska Str. 8, 31-066 Krakow, Poland

**Keywords:** anti-retinal antibodies, anti-endothelial cell antibodies, age-related macular degeneration, geographic atrophy, indirect immunofluorescence

## Abstract

*Background and Objectives*: Age-related macular degeneration (AMD) is one of the leading causes of central vision loss among elderly patients, and its dry form accounts for the majority of cases. Although several causes and mechanisms for the development and progression of AMD have previously been identified, the pathogenesis of this complex disease is still not entirely understood. As inflammation and immune system involvement are strongly suggested to play a central role in promoting the degenerative process and stimulating the onset of complications, we aimed to analyze the frequency of serum anti-retinal (ARAs) and anti-endothelial cell antibodies (AECAs) in patients with dry AMD and to determine their relationship with the clinical features of the disease, notably the area of geographic atrophy (GA). *Materials and Methods*: This study included 41 patients with advanced-stage dry AMD and 50 healthy controls without AMD, matched for gender and age. ARAs were detected by indirect immunofluorescence using monkey retina as an antigen substrate, and the presence of AECAs was determined using cultivated human umbilical vein endothelial cells and primate skeletal muscle. *Results:* ARAs were detected in 36 (87.8%) AMD patients (titers ranged from 1:20 to 1:320) and in 16 (39.0%) (titers ranged from 1:10 to 1:40) controls (*p* = 0.0000). Twenty of the forty-one patients (48.8%) were positive for AECAs, while in the control group, AECAs were present only in five sera (10.0%). The titers of AECAs in AMD patients ranged from 1:100 to 1:1000, and in the control group, the AECA titers were 1:100 (*p* = 0.0001). There were no significant correlations between the presence of AECAs and disease activity. *Conclusions*: This study demonstrates a higher prevalence of circulating AECAs in patients with dry AMD; however, no correlation was found between the serum levels of these autoantibodies and the area of GA.

## 1. Introduction

Age-related macular degeneration (AMD) is one of the most common causes of central vision loss in people over 50 years of age in developed countries, with an estimated new case rate of 0.19% per year [1]. The loss of central vision is a result of geographical atrophy (GA) occurring during the course of dry (atrophic, non-exudative) AMD or macular neovascularization (MNV) associated with wet (exudative) AMD. Dry AMD accounts for 85–90% of all cases of AMD [2]. In its advanced form, dry AMD is characterized by GA, defined as a round or oval area of sharply demarcated atrophic lesion involving photoreceptors, retinal pigment epithelium (RPE), and choroidal vessels with a diameter of at least 175 µm. GA typically begins in the perifoveal region and gradually progresses to involve the fovea over time, resulting in central scotomas and permanent loss of visual acuity. According to statistical data, over 5 million people around the world are diagnosed with GA each year and it is a significant cause of irreversible central vision loss, with prevalence in European populations increasing from 1.4% at 75 years to 11.3% in those 90 years of age or older [3,4]. The prevalence of AMD varies according to ethnicity, with non-Hispanic Caucasian Europeans having the highest burden of the disease [5]. The median time from diagnosis to central vision loss ranges from 1.4 to 2.5 years, which is connected with the onset of foveal involvement [4].

AMD appears to be a complex condition and consists of numerous processes and mechanisms, including various non-modifiable risk factors, such as age, female gender, white race, and genetic background, which affect its development and progression [6,7,8]. Over 30 identified genes are linked to a higher risk of AMD development. However, the strongest genetic risk variant for AMD is thought to be polymorphism of the complement factor H gene (Y402H) [9,10,11]. Modifiable risk factors include the following: cigarette smoking, cardiovascular diseases, high serum lipid levels, abdominal obesity, a diet with a low intake of antioxidants, and exposure to ultraviolet radiation, as well as local factors such as cataract surgery and blue irises [6,7,12,13,14,15].

There is growing evidence that one possible factor involved in the pathogenesis and progression of AMD is autoimmunity against retinal antigens and inflammatory reactions dependent on complement system activation [16,17,18,19]. In the aging process, chronic inflammation, known as para-inflammation or inflammaging, is believed to be an adaptive response of the immune system to increased oxidative stress and noxious insults so as to maintain local tissue homeostasis. In patients with AMD, this chronic inflammatory reaction becomes dysregulated and contributes to macular damage [20]. A number of publications have demonstrated that various ocular diseases in the course of which autoimmune and inflammatory components play a crucial role may be associated with the presence of serum anti-retinal antibodies (ARAs) [21,22,23,24,25,26,27,28,29,30,31]. However, the actual role played by these autoantibodies in the pathogenesis of AMD remains unclear. There is some speculation that their occurrence may be an epiphenomenon that develops in response to macular damage or, alternatively, ARAs may play a direct role in the development and progression of AMD [32,33,34,35,36,37].

Although we have already reported on the prevalence of serum anti-endothelial cell antibodies (AECAs) in patients with wet AMD [38], a review of the literature reveals that, to date, no publications have focused on the presence of circulating AECAs in the course of dry AMD. To the best of our knowledge, the present study is the first research to be conducted on the occurrence of circulating AECAs in patients with dry AMD.

The purpose of our study was to determine the prevalence of serum AECAs in patients with dry AMD in order to determine their relationship with the clinical features of the disease, notably in the area of GA.

## 2. Materials and Methods

### 2.1. Patients and Controls

Forty-one out of sixty-three consecutive patients diagnosed with GA related to dry AMD between February 2019 and December 2019 met the eligibility criteria and were enrolled in the study. The exclusion criteria included the following: other retinal diseases (MNV, macular telangiectasia, degenerative myopia), infectious or non-infectious uveitis, glaucoma, paraneoplastic retinopathies, diabetic retinopathy, systemic autoimmune comorbidities, and systemic steroid and immunosuppressive therapy. The control group comprised fifty sex- and age-matched healthy subjects, scheduled to undergo moderate senile cataract surgery with no clinical signs of AMD or any chronic eye disease. The absence of AMD in controls was determined by an ophthalmological examination and supported by the AMD classification system prepared by Ferris et al. [2]. This system is based on fundus lesions observed within 2 disk diameters of the fovea. Individuals with no drusen or pigmentary changes or with small drusen (<63 µm) in the macula were considered to have no features of AMD.

The baseline ophthalmic examination performed on both the patients and the controls included the best corrected visual acuity (BCVA) assessment with Snellen charts, as well as an anterior segment and fundus examination. Diagnoses of dry AMD were based on the presence of clinical characteristics detected by means of fundoscopy, the results of optical coherence tomography (OCT) (Topcon 3D OCT 2000, Tokyo, Japan), and fundus autofluorescence (FAF) (Heidelberg Engineering, Spectralis HRA-OCT, Heidelberg, Germany), which was used to measure the area of geographic atrophy. A color fundus image was also obtained for each patient.

This study complied with the Declaration of Helsinki. Approval by the Jagiellonian University Bioethical Committee (approval no. 1072.6120.37.2019) was obtained. All subjects provided written informed consent to participate in the study.

### 2.2. Autoantibody Assays

Circulating ARAs and AECAs were assessed using the indirect immunofluorescence (IIF) method. The principle of this two-step method is to expose a standardized antigen substrate (tissue section, cell line, or cells overexpressing a particular antigen) to a patient’s serum at an initial dilution (1:10 to 1:100), and next, if the reaction is positive, to serial dilutions of the tested serum. A secondary antibody conjugated to a fluorophore is used to detect the primary human autoantibody. IIF is a semiquantitative method and the levels of antibodies are analyzed by performing dilutions on the serum sample and reported as a titer.

From each subject of the study, 5 mL of blood from the peripheral vein was collected, clotted, and centrifuged at 3500 rpm (1970× *g*) for 10 min to recover serum. The aliquoted serum samples were stored at −80° until analysis. The collected material was examined for ARA by the IIF method based on commercially available frozen sections of normal monkey retina. To detect antigen-bound autoantibodies, a secondary goat anti-human IgA, G, M polyclonal antibody labeled with fluorescein isothiocyanate was used (Euroimmun AG, Lubeck, Germany). Serum from the patients or controls in dilutions of 1:10, 1:20, 1:40, 1:80, etc., was used to titer positive samples against ARAs, whereas a starting serum dilution of 1:10 was used to screen against AECAs, in which the human umbilical vein endothelial cells (HUVECs) and iliopsoas muscle sections served as antigen substrates (Euroimmun AG, Lubeck, Germany). All details of the IIF processing procedure, including titering, incubation, and the washing protocol, conformed to the manufacturer’s recommendations. In brief, the incubation time for the serum or the secondary antibody was 30 min. After each incubation with the diluted serum followed by the secondary antibody, the biochips were rinsed with phosphate-buffered saline and Tween 20. Samples with a positive immunological reaction to retinal vessels were screened for AECAs using IIF performed on monkey iliopsoas muscle and HUVECs. All immunohistochemical slides were evaluated using a EUROSTAR-Bluelight fluorescence microscope (Carl Zeiss Axioscope 5, Euroimmun, Germany). The IIF results were scored by two independent investigators, neither of whom were aware of the diagnosis or the stage of the disease. The results were recorded as the presence of a fluorescence signal in the layers of the retina, iliopsoas muscle, and HUVECs at the highest serum dilution at which autoantibodies were still detectable. The signal intensity of fluorescence was assessed qualitatively using a fluorescence microscope. In positive samples, the tissue or cells show a bright apple-green fluorescence with a staining pattern characteristic of the particular antigen distribution within the substrate. If the sample was negative, no clearly detectable fluorescence was observed. A commercial negative tissue control was included on each slide to eliminate the misinterpretation caused by autofluorescence of the tissue. Pictures of the microscope slides were taken for documentation purposes.

### 2.3. Statistical Analysis

A correlation between nominal variables and ordinal variables indicated an association between the variables, while the significance level was evaluated using the chi-square test for two-way tables. A nonparametric Spearman’s rank correlation coefficient was used to assess the statistical dependence between discrete and continuous variables. Student’s *t* distribution or nonparametric Mann–Whitney U tests were used to compare the mean values in two groups. A type I statistic error *p*-value of <0.05 was deemed significant.

## 3. Results

### 3.1. Epidemiology and Characteristics of Patients with Dry AMD and a Control Group

The study group included 41 patients (82 eyes) with dry AMD. This total included 15 males (36.6%) and 26 females (63.4%) aged 61–90 years (mean: 76.3 years). The control group consisted of 20 males (40%) and 30 females (60%) aged 59–86 years (mean: 71.9 years). No statistical differences in terms of age or gender distribution were noted among patients with AMD and the controls. No significant statistical differences were observed between the study and control groups in terms of the prevalence of cardiovascular diseases and hyperlipidemia. Table 1 shows the clinical characteristics of patients with dry AMD and the subjects comprising the control group.

The baseline BCVA ranged from counting fingers to 0.8 on the Snellen chart. Fundoscopy revealed various distinctive changes observed in dry AMD including the following: areas of chorioretinal atrophy observed in 80 eyes and large drusen with changes in the retinal pigment epithelium (RPE) observed in 2 eyes. OCT scans revealed typical findings for dry AMD; large drusen or atrophy of the outer retinal layers involving photoreceptors, Bruch’s membrane, and choriocapillaris.

According to the FAF examination, the mean area of geographic atrophy ranged from 0.24 mm^2^ to 29.12 mm^2^ (mean: 9.81 mm^2^).

### 3.2. Serum Anti-Retinal Antibodies in Dry AMD Patients and in a Control Group

**Figure 1:**
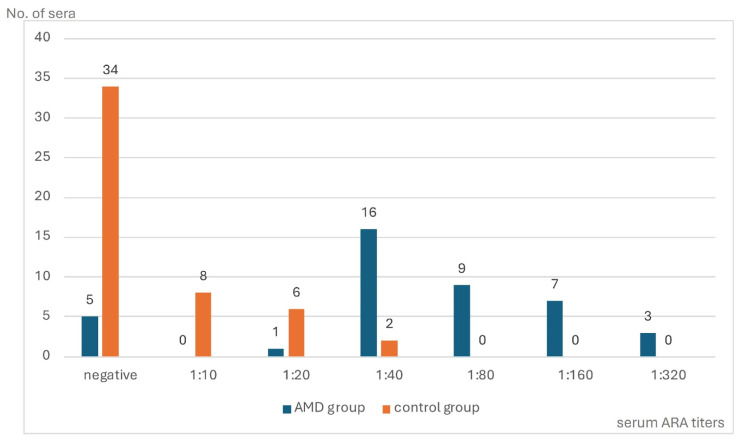
The distribution of circulating anti-retinal antibody (ARA) titers in patients with dry age-related macular degeneration (AMD) and in a control group.

The IIF test performed on the monkey retina revealed circulating ARAs in 36 (87.8%) of the 41 AMD patients (range from 1:20 to 1:320) as well as in 16 of the 50 (39.0%) (range from 1:10 to 1:40) controls (*p* = 0.0000). A comparison of the distribution and titers of the ARAs in the sera of both the AMD patients and the control group is presented in Figure 1.

Interestingly, in patients with dry AMD, three types of retinal staining on monkey retina were detected in the IIF test, while the control sera revealed two staining patterns of reactivity with the retinal tissue. Differences in ARA types observed in the AMD and control groups were statistically significant in cases where there was a positive reaction to cones and rods (*p* = 0.0000 and *p* = 0.0001, respectively), while immunofluorescence within the cytoplasmic components of the retinal nuclear layer cells showed no statistical differences between the two analyzed groups. Table 2 presents the distribution of ARAs in the serum of patients with dry AMD and controls according to the staining pattern and titer.

**Figure 2:**
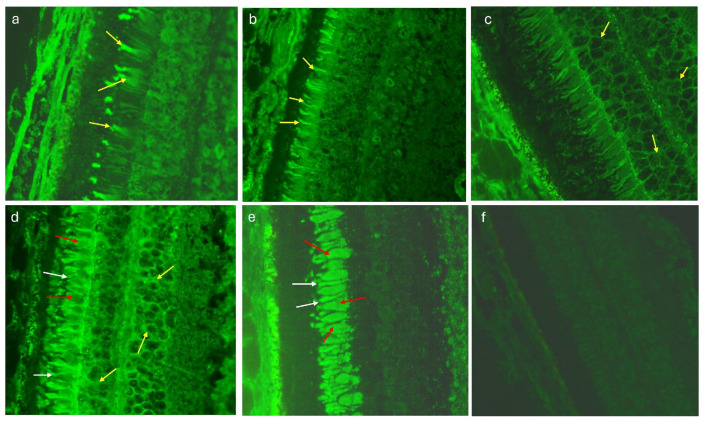
Monkey retina—indirect immunofluorescence test (IIF): (**a**) a positive staining of cones (yellow arrows), magnification 400×; (**b**) a positive staining of rods (yellow arrows), magnification 400×; (**c**) a positive staining of cytoplasmic components of both retinal nuclear layers (yellow arrows), magnification 400×; (**d**) a combined positive reaction within cones (red arrows), rods (white arrows) and cytoplasmic components of both retinal nuclear layers (yellow arrows), magnification 400×; (**e**) a positive reaction within photoreceptors: cones (red arrows) and rods (white arrows), magnification 400×; (**f**) a negative control, magnification 400×.

The sera of 31 (75,6%) patients with dry AMD showed more than one immunofluorescence type, while in the control group, complex ARA types were observed in only 4 (8%) cases (*p* = 0.0000). Figure 2 presents various immunofluorescence pattern reactions of AMD patients’ sera with monkey retina.

### 3.3. Anti-Endothelial Cell Antibodies in Patients with Dry AMD and in a Control Group

**Figure 3:**
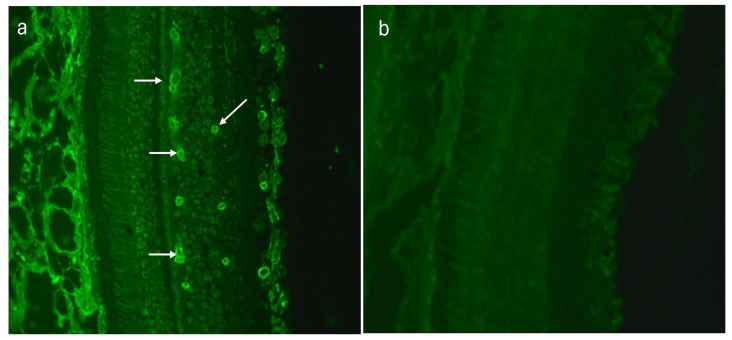
(**a**) Indirect immunofluorescence (IIF) test performed on monkey retina revealed positive retinal vessel pattern staining (white arrows), magnification 200×; (**b**) monkey retina—negative control, magnification 200×.

The sera of 20 (48.8%) AMD patients revealed a highly distinctive tubular arrangement in the fluorescence signal recorded on monkey retina sections within the retinal vessels (Figure 3a,b). In the control group, this type of immunofluorescence was observed in only five (10%) sera. This difference was statistically significant (*p* = 0.0001). 

**Figure 4:**
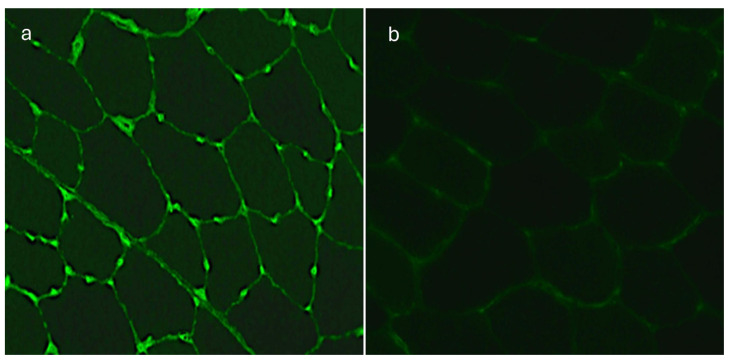
(**a**) Iliopsoas muscle—indirect immunofluorescence (IIF) test demonstrates a positive reaction with serum of a patient with dry AMD; immunofluorescence of endothelial cells of muscle vessels is observed, magnification 400×. (**b**) Iliopsoas muscle—a negative reaction with a control serum, magnification 400×.

**Figure 5:**
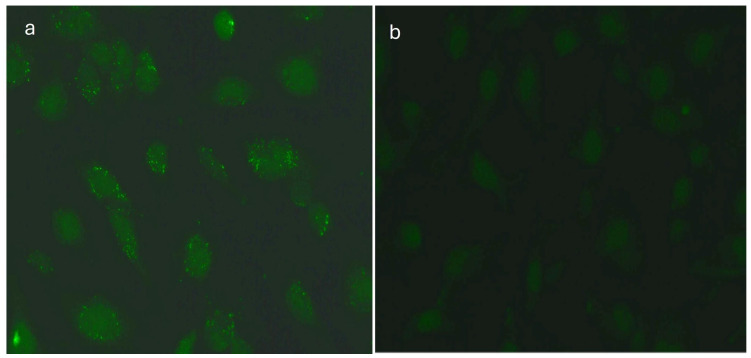
(**a**) Human umbilical vein endothelial cells (HUVECs)—indirect immunofluorescence (IIF) test shows a positive reaction with AMD patient serum; immunofluorescence of endothelial cells is present, magnification 400×. (**b**) Human umbilical vein endothelial cells (HUVECs)—a negative reaction with a control serum, magnification 400×.

Since this type of immunofluorescence suggested the presence of anti-endothelial specific autoantibodies, we repeated the assay with a primary human cell line taken from the umbilical vein (HUVEC) and skeletal iliopsoas muscle sections of monkeys as the substrates. The fluorescence signal located between the fibers of the transversal sections of the muscle had a characteristic pattern corresponding to striated muscle vessels. Besides endothelial cells, the HUVEC cellular substrate has no other epitopes. The presence of autoantibodies identified in IIF tests performed on monkey iliopsoas muscle and HUVECs confirmed the presence of AECAs in 20 (48.8%) patients with dry AMD in titers ranging from 1:100 to 1:1000. All 20 (48.8%) sera of the AMD patients showed a positive reaction with the iliopsoas muscle and 13 (31.7%) of them with HUVECs, thus demonstrating an immunofluorescence pattern typical for AECAs (Figure 4 and Figure 5).

Only five (10.0%) of the control sera showed a positive immunofluorescence pattern reaction to retinal vessels in the IIF test performed on normal monkey retina in titers ranging from 1:10 to 1:80. Tests carried out on monkey iliopsoas muscle and HUVECs confirmed the presence of circulating AECAs in all five (10.0%) control sera at titer 1:100.

Table 3 presents the distribution of AECAs in the sera of both patients with dry AMD and controls according to the immunofluorescence staining pattern and titer tests.

### 3.4. Circulating Anti-Retinal and Anti-Endothelial Cell Antibodies and Clinical Features of Dry AMD

**Figure 6:**
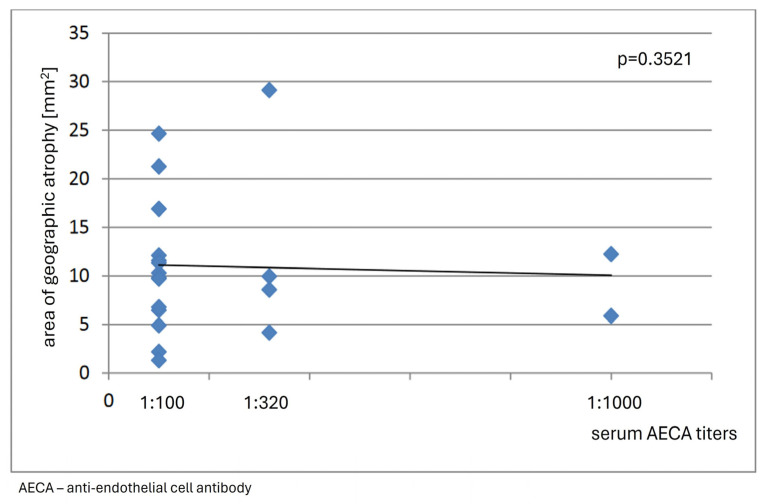
The graph shows no association between the area of geographic atrophy and serum anti-endothelial cell antibody (AECA) titers in patients with dry age-related macular degeneration.

No correlation was found between immunofluorescence staining patterns of reactivity against retinal tissue, complexity and serum levels of circulating ARAs, and clinical features of dry AMD in the analyzed group of patients, nor did the observations reveal any association between serum levels of circulating AECAs and the area of GA (*p* = 0.3512) (Figure 6).

The simultaneous occurrence of circulating ARAs and AECAs did not differ from cases in which the sera were shown to be positive only for ARAs or AECAs in terms of the geographic atrophy area. The study showed no association between circulating ARAs and AECAs in the analyzed group of patients with dry AMD.

## 4. Discussion

GA is a multifactorial disease in which intrinsic and extrinsic factors result in RPE dysfunction. With age, RPE dysfunction progresses, leading to drusen and lipofuscin deposition [11,39]. Drusen and lipofuscin components, as well as other oxidative stress products, such as advanced glycation end products, have been reported to induce inflammation through multiple pathways, including via the complement cascade [39,40,41,42]. When regulatory components in these pathways are compromised, especially by genetic risk factors, initiation of chronic inflammation (para-inflammation) may lead to damage and, ultimately, retinal cell death, which is a typical feature of GA [11,42]. Although the mechanism of late-stage dry AMD is not fully understood, inflammation and immune dysregulation are believed to play a significant role in its pathogenesis [43]. Pathological mechanisms initiating excessive apoptosis of photoreceptors in the course of AMD have also been implicated as a potential pathogenetic factor in this multifactorial condition [44].

AMD is categorized as a neurodegenerative disorder, and its pathogenesis bears considerable similarities to Alzheimer’s disease. Both diseases are strongly associated with increased age. In addition, oxidative stress, inflammation, and complement activation are believed to be associated with both diseases [45,46]. There have been reports of a higher prevalence of Alzheimer’s disease in patients with AMD, and it has even been hypothesized that AMD and Alzheimer’s disease may be two variants of the same disorder [46,47,48]. Thus, AMD is not simply indicative of ocular disease, but may also signal systemic processes of accelerated aging.

Despite a number of studies analyzing the associations between AMD and cardiovascular diseases, such relationships are inconsistent and remain controversial. There are reports describing significant relationships between the occurrence of these conditions and AMD; however, other studies have reported inverse associations between AMD and cardiovascular comorbidities, suggesting their protective effect [49,50,51,52]. The majority of studies suggest a significant association between the presence of atherosclerotic plaques and the development of AMD; however, some of them describe no such association [49,53]. Several reports have found significant associations between stroke and systemic hypertension and AMD, while others have reported no significant relationships between these systemic conditions and AMD [54,55]. Similar to other cardiovascular diseases, later studies showed different results regarding the association between serum lipids levels and AMD. Our study demonstrated no significant differences between patients with AMD and a control group in terms of cardiovascular diseases. Regarding the relationship between AMD and systemic conditions, the observations show that the incidence of AMD is lower in patients with diabetes than in the general population. However, other reports have suggested that the presence of diabetic retinopathy may be a risk factor for AMD [56].

In the present study, circulating ARAs were detected at significantly higher frequencies and at higher titers in patients with GA compared with the controls. The literature contains only limited data on the prevalence of serum ARA in patients with dry AMD; however, most of those studies reported autoantibody analysis for both wet and dry AMD [33,57]. Recently, more studies have demonstrated the presence of circulating ARAs in patients with exudative AMD. The results of these studies revealed the presence of ARAs in between 46% and 95.9% of patients with exudative AMD [32,33,36,58,59]. This discrepancy in the occurrence of circulating ARAs may be due to the use of different diagnostic methods and different dilutions of the tested sera. Additionally, it may also be related to the heterogeneity of the studied groups, as well as differences in the type and stage of the disease. Previous studies revealed associations between ARA serum levels and the stage of exudative AMD. Moreover, treatment with intravitreal VEGF inhibitors led to a decrease in the titers of these autoantibodies [36]. Bearing in mind the fact that exudative and dry AMD are two forms of the same condition, it seems that the effect of ARAs is still insufficiently understood in patients with dry AMD. Korb et al. [37] described ARAs in patients with dry AMD and found that these autoantibodies occurred more frequently when compared to a healthy population. However, this study did not analyze the possible association of this factor with the stage of the disease, and thus, it is difficult to compare those results with our observations. In our study, serum ARAs were detected more frequently in individuals with GA than in controls without AMD, suggesting their involvement in the progression of the disease. The results of this study may support the hypothesis that circulating ARAs may play some role in the pathogenesis of GA. Since it has been shown that circulating ARAs can appear 3 to 15 years prior to the clinical manifestation of the disease, this seems a reasonable topic for further research [60,61].

To the best of our knowledge, the current study is the first to report the presence of circulating AECAs in patients with dry AMD. We found significantly elevated levels of AECAs in the serum of patients with GA. Studies on the association between circulating AECAs and dry AMD appear to be limited. Machalinska et al. [62] reported circulating endothelial cells (CECs) in patients with wet and dry AMD but did not assess serum AECA levels. Their study indicated that AMD is accompanied by endothelial dysfunction. Increased serum CEC counts in patients with AMD reflect severe vascular abnormalities and may play a role in the pathogenesis of the disease. The authors also emphasized the need to search for a common pathological mechanism for AMD and systemic vascular diseases [62].

AECAs were first described by Lindqvist and Osterland in 1971 [63]. These autoantibodies were reported in several autoimmune diseases associated with vasculitis, such as systemic lupus erythematosus (SLE), systemic scleroderma (SSc), rheumatoid arthritis, Kawasaki disease, granulomatosis with polyangiitis (GPA), multiple sclerosis, and diabetes mellitus [23,64,65]. However, the presence of AECAs is not a disease-specific marker, although it can play an important role in monitoring disease activity, and can also be used to assess the risk of recurrence or complications [65]. In patients with SSc, while no correlation was observed with AECAs and SSc activity, the authors suggested that the presence of AECAs might indicate vascular complications in SSc [64]. In some reports, it has been claimed that AECAs may be a good predictor of relapses in patients with small vessel vasculitis [66].

It has been hypothesized that AECAs may occur in response to endothelial cell injury. However, it is also possible that they themselves are characterized by cytotoxic activity, and their presence may be inherent in the destructive inflammatory process [60,65,67]. The cytotoxic activity of AECAs can affect endothelial cells through complement-dependent mechanisms or as an antibody-dependent cell-mediated response [67]. Some publications have suggested that by inducing the overexpression of adhesion molecules (including selectins, intercellular adhesion molecule-1, and vascular cell adhesion molecule-1) and the production of cytokines and chemokines, AECAs may cause endothelial damage through their adhesion to endothelial cells. An autoimmune basis of vasculitis is therefore suggested [65,68,69]. AECAs were shown to induce apoptosis of the progenitors of bone marrow endothelial cells, which in turn leads to disturbances in endothelial regeneration and the healing of vascular lesions [70]. A number of studies have also indicated that AECAs may exert pathogenic effects by activating endothelial cells to induce a procoagulant phenotype and subsequent thrombosis [69,71,72,73].

The results of this study revealed significant differences in the occurrence of circulating AECAs in patients with dry AMD compared with the controls; these autoantibodies were detected more frequently and at higher titers in the former group. Our previous study on patients with wet AMD also showed significant differences in the prevalence of AECAs between the study group and healthy subjects, while no association was noted between disease progression and average serum AECA titers [38]. These observations are in accordance with the results reported in the present research, which showed no association between AECA titers and the clinical activity of the disease assessed as an area of GA.

The above-described mechanisms characterizing the initiation and progression of tissue damage in connection with the presence of circulating AECAs cannot be excluded as factors in the course of dry AMD.

## 5. Conclusions

This study demonstrated a higher prevalence of circulating AECAs in patients with dry AMD; however, no correlation was observed between serum levels of these autoantibodies and the clinical feature of the disease, assessed as the area of GA.

These findings may suggest that AMD is an immune-mediated inflammatory disease. However, further studies are needed to determine whether AECAs are involved in the pathogenesis of dry AMD or whether they occur secondary to endothelial cell damage in the course of the disease.

## Figures and Tables

**Table 1:** Epidemiologic and clinical characteristics of patients with dry age-related macular degeneration (AMD) and a control group.

Characteristics	AMD Group	Control Group	*p* Value
	*n* = 41	*n* = 50	
Sex			0.739
Females	26 (63.4%)	31 (62.0%)	
Males	15 (36.6%)	19 (38.0%)	
Age	61–90 years (mean: 76.3 years)	59–86 years (mean: 71.9 years)	0.077
Arterial hypertension	20 (48.8%)	11 (22.4%)	0.5324
Ischemic heart disease	6 (14.6%)	3 (6.1%)	0.1931
Atherosclerosis	5 (12.2%)	1 (2.0%)	0.0678
Hyperlipidemia	4 (9.8%)	3 (6.1%)	0.6109

**Table 2:** Distribution of serum anti-retinal antibodies in patients with dry age-related macular degeneration (AMD) and controls according to the titer and immunofluorescence staining pattern on retinal tissue.

Type of Immunofluorescence Staining Pattern	Study Group		Titre						*p* Value
		Negative	1:10	1:20	1:40	1:80	1:160	1:320	
Cones, *n* (%)	AMD *n* = 41	17 (41.5)	0 (0)	1 (2.4)	13 (31.7)	5 (12.2)	3 (7.3)	2 (4.9)	0.0000
	Control *n* = 50	50 (100)	0 (0)	0 (0)	0 (0)	0 (0)	0 (0)	0 (0)	
Rods, *n* (%)	AMD *n* = 41	20 (48.8)	0 (0)	0 (0)	2 (4.8)	10 (24.4)	7 (17.1)	2 (4.8)	0.0005
	Control *n* = 50	34 (68.0)	7 (14.0)	7 (14.0)	2 (4.0)	0 (0)	0 (0)	0 (0)	
Cytoplasmic components of retinal nuclear layer cells, *n* (%)	AMD *n* = 41	34 (82.9)	0 (0)	0 (0)	5 (12.2)	1 (2.4)	1 (2.4)	0 (0)	0.3230
	Control *n* = 50	34 (96)	0 (0)	1 (2)	1 (2)	0 (0)	0 (0)	0 (0)	
Retinal vessels, *n* (%)	AMD *n* = 41	21 (51.2)	0 (0)	0 (0)	9 (22)	5 (12.2)	4 (9.8)	2 (4.9)	0.0001
	Control *n* = 50	45 (90)	3 (6)	2 (4)	0 (0)	0 (0)	0 (0)	0 (0)	

**Table 3:** Distribution of anti-endothelial cell antibodies (AECAs) in the sera of patients with dry age-related macular degeneration (AMD) and controls according to the titer and immunofluorescence staining pattern on retinal tissue.

Type of Immunofluorescence Staining Pattern	Study Group	Titer				*p* Value
		Negative	1:100	1:320	1:000	
Iliopsoas muscle, *n* (%)	AMD *n* = 41	21 (51.2)	14 (34.1)	4 (9.8)	2 (4.9)	0.0000
	Control *n* = 50	45 (90)	5 (10)	0 (0)	0 (0)	
HUVECs, *n* (%)	AMD *n* = 41	28 (68.3)	8 (19.5)	3 (7.3)	2 (4.9)	0.0001
	Control *n* = 50	45 (90)	5 (10)	0 (0)	0 (0)	

HUVECs—human umbilical vein endothelial cells.

## Data Availability

Data supporting the findings of this study are available upon request from the corresponding author.
